# Supplementary material for: GLI2 and FLNB Define Periocular Morphoeic Basal Cell Carcinoma
Source: Int J Mol Sci. 2025 Nov 25;26(23):11377. doi: 10.3390/ijms262311377 (PMC12692270; doi:10.3390/ijms262311377)
Supplement: Supplementary file 1 [file ijms-26-11377-s001.zip › Supplementary Table S1.pdf]

Gene	Freq	Driver	q-value	Gene	Freq	Driver	q-value
PTCH1	8	K, HCD	1.06E-10	*CHKB*	2	CD	0.04
USP4	4		1.91E-07	*FNIP1*	2		0.04
FLNB	5	CD	1.51E-8	*SMARCA4*	4	K, HCD	0.04
CHD3	2	I, CD	1.41E-05	*LAMA2*	5	I	0.06
EPHA3	3	CD	0.01	*ARHGAP35*	2	I	0.06
NFS1	3		0.04	*SYNE1*	6		0.06
EFTUD2	2	I, CD	0.04	*SRPX2*	2		0.06
FLNC	4	CD	0.04	*TMEM87B*	2		0.06
HECTD4	4		0.04	*ITGA1*	2		0.07

Supplementary Table S1. Intogen derived drivers in mBCC. Top 18 driver genes arranged using Intogen and a fm-bias (q-value) value <0.1. I, Intogen cancer driver. CD, Connected to a known driver; HCD, high confidence driver; K, known driver, Freq, frequency.
