# Supplementary material for: GLI2 and FLNB Define Periocular Morphoeic Basal Cell Carcinoma
Source: Int J Mol Sci. 2025 Nov 25;26(23):11377. doi: 10.3390/ijms262311377 (PMC12692270; doi:10.3390/ijms262311377)
Supplement: Supplementary file 1 [file ijms-26-11377-s001.zip › Supplementary Table S10.pdf]

**Sample**	**Age**	**Gender**	**Location**	**Sample**	**Age**	**Gender**	**Location**
**M1**	85	M	RLL	**Nod1**	70	M	LLL
**M2**	47	F	LLL	**Nod2**	80	M	RLL
**M3**	78	F	LLL	**Nod3**	71	M	RMC
**M4**	71	M	LMC	**Nod4**	72	F	LLL
**M5**	83	F	LMC	**Nod5**	46	F	RLL
**M6**	61	M	RLL	**Else1**	74	M	Chest
**M7**	71	F	RMC	**Else2**	89	M	Chest
**M8**	78	F	RUL	**Else3**	83	M	Forearm
**M9**	62	F	RUL	**Else4**	87	M	Forearm
**M10**	87	F	RMC	**Else5**	52	M	Shin

Supplementary Table S 10. Clinical features of basal cell carcinoma (BCC) patients. Summary of the clinical features for each BCC histological subtype. M, morphoeic BCC, Nod, nodular BCC, Else, non-periocular nodular BCC found outside the H-zone; LLL, left lower eyelid; RLL, right lower eyelid, LUL, left upper eyelid; RUL right upper eyelid.
