# Supplementary material for: GLI2 and FLNB Define Periocular Morphoeic Basal Cell Carcinoma
Source: Int J Mol Sci. 2025 Nov 25;26(23):11377. doi: 10.3390/ijms262311377 (PMC12692270; doi:10.3390/ijms262311377)
Supplement: Supplementary file 1 [file ijms-26-11377-s001.zip › Supplementary Table S11.pdf]

FLNB:5′-TGATCTATGTGCGCTTCGGT-3′(forward), 5′-TCGGTCACCCAGGGC-3′(reverse). *HECTD4*:5′-GCATCTTGTGGGTCCAGTGA-3′(forward), 5′-CTTCGCTCCTGGTGTAAGGT-3′(reverse).
GAPDH: 5′-CGCTCTCTGCTCCTCCTGTT-3′(forward), 5′-CCATGGTGTCTGAGCGATGT-3′(reverse).
β-actin:5′- ACTCTTCCAGCCTTCCTTC-3′(forward), 5′-GATGTCCACGTCACACTTC-3′(reverse). *ECAD*: 5' CGGACGATGATGTGAACACC 3' (forward), 5' TTGCTGTTGTGCTTAACCCC 3' (reverse).
GLI1: 5' GAAGACCTCTCCAGCTTGGA 3' (forward), 5' GGCTGACAGTATAGGCAGAG 3' (reverse).
GLI2: 5' TGGCCGCTTCAGATGACAGATGTTG 3' (forward), 5' CGTTAGCCGAATGTCAGCCGTGAAG 3' (reverse).
IRF2: 5' GCCTCAGAACGGACGAGATA 3' (forward), 5' ATAGGAAGAGAGGCTGGGGA 3' (reverse).
IRF3: 5' AAGAGGCTCGTGATGGTCAA 3' (forward), 5' CTTGTACTGGTCGGAGGTGA 3' (reverse).
LOXL2: 5'AAGCTCAGGAGGAAAGGGAC 3' (forward), 5' GCATTTGTCCACGTGTCACT 3' (reverse).
MYCN: 5' CCAAGGCTGTCACCACATTC 3'(forward), 5' GCATCCTCACTCTCCACGTA 3' (reverse).
NCAD: 5' CGGTTTCATTTGAGGGCACA 3' (forward), R-5' TTGGAGCCTGAGACACGATT 3' (reverse).
NFIB: 5' CCTCCTCGTGTACCAGGAAG 3' (forward), 5' AGCACACAAAGGCTGAACAG 3' (reverse).
PLAT: 5' TGCTTGGTCTGATGGGCTAA 3' (forward), 5' GTCCACGTCTCACCCAGTTA 3' (reverse).
PPM1D: 5' TGGGTGAGCATGGACAATCT 3' (forward), 5' GGTGGTGTAGAACATGGGGA 3' (reverse).
PTCH1: 5' ACTCGCCAGAAGATTGGAGA 3' (forward), 5' TCCAATTTCCACTGCCTGTT 3' (reverse).
SLC7A11: 5' TCCGATCTTTGTTGCCCTCT 3' (forward), 5' GACTGTCGAGGTCTCCAGAG 3' (reverse).
SMO: 5' GGCTGCTGAGTGAGAAG 3' (forward), 5' CTGGTTGAAGAAGTCGTAGAAG 3' (reverse).
STAT6: 5' AAGAGCACAGGTTAGGGCAT 3' (forward), 5' TAACCACATGTCCAGACCCC 3' (reverse).
TGF-β2: 5' AGACAGCAGAGCGAGAGC 3'(forward), 5' GTAGGCACTTGCCACCCTAA 3' (reverse).
TRIM22: 5' TAGCCCTTGTGCTGAGACTC 3' (forward), 5' CCTTCCTTGCAGACCTCAGA 3' (reverse).
TRIP4: 5' CCAACATGTACCAGTCCCCT 3' (forward), 5' CCACCATCAAAGCCTTCCTG 3' (reverse).
ZNF114: 5' ACAGAGACGTGATGCTGGAA 3' (forward), 5' TCCTCTGTTTTGGGGCATCT 3' (reverse).

Table S11. Primer sequence of *FLNB*, *HECTD4*, *GAPDH* and *β-actin.*
