# Supplementary material for: GLI2 and FLNB Define Periocular Morphoeic Basal Cell Carcinoma
Source: Int J Mol Sci. 2025 Nov 25;26(23):11377. doi: 10.3390/ijms262311377 (PMC12692270; doi:10.3390/ijms262311377)
Supplement: Supplementary file 1 [file ijms-26-11377-s001.zip › Supplementary Table S2.pdf]

Gene	Non-silent	P Value	Gene	Non-silent	P Value
PTCH1	8	1.61E-07	*SPINK9*	1	3.04E-02
C6	7	4.02E-02	*IL17F*	1	3.06E-02
POM121L12	6	1.46E-02	*EDDM3B*	1	3.07E-02
CCDC108	4	1.12E-02	*KCNE4*	1	3.29E-02
SRPX2	3	2.65E-02	*HPRT1*	1	3.43E-02
ARL16	3	3.89E-02	*RPL11*	1	3.45E-02
PIN4	3	4.07E-02	*STARD6*	1	3.46E-02
OR5M10	3	4.24E-02	*MED31*	1	3.50E-02
FBXO15	2	1.33E-02	*NMS*	1	3.64E-02
OR5H2	2	1.84E-02	*TCEAL6*	1	3.77E-02
OR56A3	2	2.42E-02	*NKIRAS1*	1	3.78E-02
TNP2	2	2.55E-02	*MRPL24*	1	4.19E-02
NOXA1	2	2.62E-02	*B9D2*	1	4.32E-02
H3F3C	2	2.98E-02	*TNFAIP6*	1	4.40E-02
SIGLEC10	2	3.95E-02	*OR5M3*	1	4.54E-02
ACVR1	2	4.03E-02	*GNGT2*	1	4.55E-02
UTS2	2	4.45E-02	*BAX*	1	4.72E-02
KRTAP12-3	1	1.99E-02	*STARD3NL*	1	4.85E-02
PAGE1	1	2.17E-02	*STX1A*	1	4.98E-02
DPPA5	1	2.27E-02			

Supplementary Table S2. Drivers in mBCC using MutSigCV. Top driver genes using MutSigCV taking into account non-silent frequency and ordered according to P value with a P<0.05.
