# Supplementary material for: GLI2 and FLNB Define Periocular Morphoeic Basal Cell Carcinoma
Source: Int J Mol Sci. 2025 Nov 25;26(23):11377. doi: 10.3390/ijms262311377 (PMC12692270; doi:10.3390/ijms262311377)
Supplement: Supplementary file 1 [file ijms-26-11377-s001.zip › Supplementary Table S3.pdf]

Gene	Freq	Driver	q-value
PTCH1	8	K, HCD	0
TP53	7	K, HCD	2.7E-10
TRIP4	4	CD	4.2E-03
MTR	2		5.2E-03
ARHGAP35	3	I, CD	5.2-03
MYCN	4	K, HCD	0.01
NARS	2		0.03
ZEB1	4	CD	0.04
PAM	4		0.04
SYNE1	8		0.04
NHSL1	4		0.04
PTPRO	3	CD	0.04
ITGAL	3	CD	0.05
RAB14	2	CD	0.05
PCLO	7	CD	0.05
WDTC1	2		0.05
ABCC9	*3*		0.07
KDR	*4*	CD	0.07
ARID1A	*2*	CD	0.07
TANC1	*2*		0.07
KANSL1	*2*		0.07
TAF4B	*2*		0.07
FADS1	*2*		0.07
IRF2	*2*	I	0.07
PPM1D	*2*	I	0.07
PGM2L1	*2*		0.07
DMXL2	*4*		0.07
EIF2C3	*2*	I	0.07
MAST4	*5*		0.09
CASP8	*2*	I, CD	0.09
BCL11A	*4*	CD	0.09
CELSR1	*3*		0.09
NDST1	*2*		0.09

Supplementary Table S3. Intogen derived drivers in nodBCC. Top 33 driver genes arranged using Intogen and a fm-bias (q-value) value <0.1. Figures are rounded up to 2 decimal places except for the top five. I, Intogen cancer drivers. CD, Connected to a known driver; HCD, high confidence driver; K, known driver, Freq, frequency.
