# Supplementary material for: GLI2 and FLNB Define Periocular Morphoeic Basal Cell Carcinoma
Source: Int J Mol Sci. 2025 Nov 25;26(23):11377. doi: 10.3390/ijms262311377 (PMC12692270; doi:10.3390/ijms262311377)
Supplement: Supplementary file 1 [file ijms-26-11377-s001.zip › Supplementary Table S4.pdf]

Gene	Non-silent	P Value
TP53	13	6.23E-05
PTCH1	9	1.92E-04
GABRA6	5	2.85E-03
OR52J3	4	3.14E-03
TSGA13	3	3.38E-03
TRIM39	3	4.18E-03
C10orf82	3	4.72E-03
FADS1	2	8.12E-03
PPM1D	2	1.06E-02
OR5J2	6	1.08E-02
ADAM29	7	1.14E-02
DYX1C1	4	1.29E-02
NTSR2	4	1.36E-02
S1PR4	3	1.66E-02
GNG4	1	1.68E-02
POMC	2	1.76E-02
KCNQ5	5	1.83E-02
SLC4A10	4	1.86E-02
STOML1	2	1.95E-02
GPR20	2	1.96E-02
XKR9	2	2.02E-02
LSM6	1	2.04E-02
IGSF5	4	2.04E-02
PENK	2	2.10E-02
OR10H2	2	2.21E-02
ART3	3	2.31E-02
YIPF7	3	2.34E-02
TAF4B	2	2.51E-02
CXorf27	1	2.60E-02
PRRX1	4	2.74E-02
GPR1	2	2.75E-02
HOXC13	3	2.84E-02
OTX2	2	2.89E-02
CTSG	2	2.96E-02
HIGD1C	2	3.03E-02
S100A7A	3	3.04E-02
RNF7	1	3.10E-02
CRYBG3	4	3.17E-02
KCNA3	4	3.19E-02
PRB2	5	3.28E-02

Gene	Non-silent	P Value
SCG2	6	3.35E-02
COCH	4	3.36E-02
BSPH1	1	3.37E-02
LMNB2	2	3.39E-02
FAM26D	2	3.46E-02
RBM18	1	3.46E-02
TPD52L3	2	3.55E-02
GPR123	3	3.60E-02
FAM71A	4	3.62E-02
GTF2A2	2	3.71E-02
MED31	1	3.89E-02
C11orf31	1	3.95E-02
CDH10	4	4.10E-02
C8orf33	1	4.15E-02
ADIG	2	4.23E-02
VEPH1	2	4.26E-02
FGF6	3	4.27E-02
FKBP1B	3	4.34E-02
DCAKD	1	4.35E-02
CPN2	3	4.39E-02
CLECL1	1	4.40E-02
C6orf118	8	4.44E-02
LRRC36	5	4.51E-02
C1orf27	2	4.52E-02
SCN10A	9	4.55E-02
PRSS35	5	4.56E-02
UCHL3	1	4.62E-02
BMP6	4	4.64E-02
SH3KBP1	3	4.69E-02
TRIP4	4	4.70E-02
TIMM50	5	4.72E-02
CD59	2	4.74E-02
NRL	2	4.78E-02
LEP	1	4.78E-02
ZNF302	2	4.83E-02
C16orf45	3	4.93E-02
CD2	4	4.95E-02
TRIT1	4	4.97E-02
TP53	13	6.23E-05

Supplementary Table S4. Drivers in nodBCC using MutSigCV. Top driver genes using MutSigCV taking into account non-silent frequency and ordered according to P value with a P<0.05.
