# Supplementary material for: GLI2 and FLNB Define Periocular Morphoeic Basal Cell Carcinoma
Source: Int J Mol Sci. 2025 Nov 25;26(23):11377. doi: 10.3390/ijms262311377 (PMC12692270; doi:10.3390/ijms262311377)
Supplement: Supplementary file 1 [file ijms-26-11377-s001.zip › Supplementary Table S5.pdf]

Supplementary Table S5A. Percentage of the genome which were targeted by copy number a

**Sample**	**Percentage**		
mBCC1	6.53%	mean mBCC	8.60%
mBCC2	28.80%		
mBCC3	12.45%		
mBCC4	12.72%		
mBCC5	9.80%		
mBCC6	4.95%		
mBCC7	5.90%		
mBCC8	0.00%		
mBCC9	4.81%		
mBCC10	0.00%		
nodBCC1	11.91%	mean nodBC	9.19%
nodBCC2	0.20%		
nodBCC3	19.50%		
nodBCC4	4.98%		
nodBCC5	6.75%		
nodBCC6	10.33%		
nodBCC7	5.65%		
nodBCC8	16.09%		
nodBCC9	9.12%		
nodBCC10	7.40%		

py number aberrations and CN-LOH events

Supplementary Table S5B. Identified copy number aberrations (gain or loss) and copy neutral -

Sample	chr	start	end	size	aberrations
mBCC1	1	762273	14220186	13457913	CN_LOH
mBCC1	17	5952	18380168	18374216	loss
mBCC1	17	19186272	81083603	61897331	gain
mBCC1	4	164085425	189065104	24979679	loss
mBCC1	9	69002294	141070335	72068041	loss
mBCC2	1	144930596	147806559	2875963	gain
mBCC2	1	196227526	208390469	12162943	gain
mBCC2	10	282897	38978124	38695227	CN_LOH
mBCC2	whole chr14			107349540	gain
mBCC2	17	19213335	81083660	61870325	gain
mBCC2	whole chr2			243199373	gain
mBCC2	whole chr20			63025520	gain
mBCC2	whole chr6			171115067	gain
mBCC2	9	286491	69002423	68715932	gain
mBCC2	9	69067824	141070335	72002511	CN_LOH
mBCC3	chr1q			123856422	gain
mBCC3	whole chr6			171115067	gain
mBCC3	9	72472831	141107536	68634705	Loss
mBCC4	whole chr2			243199373	gain
mBCC4	9	71081166	141107573	70026407	CN_LOH
mBCC4	18	19648972	77960841	58311869	CN_LOH
mBCC5	10	48944355	135473125	86528770	loss
mBCC5	16	70863630	71196510	332880	gain
mBCC5	19	281360	15587345	15305985	loss
mBCC5	19	17544165	22498943	4954778	gain
mBCC5	21	9825747	9912364	86617	gain
mBCC5	3	9719864	58389462	48669598	loss
mBCC5	7	35378	57832955	57797577	loss
mBCC5	9	68745714	141107579	72361865	loss
mBCC6	1	186567252	217824575	31257323	CN_LOH
mBCC6	1	217856787	223799857	5943070	loss
mBCC6	1	223799871	249110906	25311035	CN_LOH
mBCC6	16	70883543	71196587	313044	gain
mBCC6	19	281360	5708484	5427124	CN_LOH

mBCC6	21	32597972	48069379	15471407	CN_LOH
mBCC6	9	45372235	69847032	24474797	CN_LOH
mBCC6	9	70999315	107267304	36267989	Loss
mBCC7	1	145608025	249230955	103622930	gain
mBCC7	9	72472831	141107536	68634705	Loss
mBCC8	none				
mBCC9	1	13209	36565832	36552623	loss
mBCC9	9	117745	35162457	35044712	gain
mBCC9	9	72333169	141107548	68774379	loss
mBCC10	none				
nodBCC1	1	142535976	249145072	106609096	gain
nodBCC1	whole chr6			171115067	gain
nodBCC1	9	71114312	141070347	69956035	loss
nodBCC2	10	37433892	42600228	5166336	loss
nodBCC2	16	46386286	46435445	49159	loss
nodBCC2	4	49094054	49659658	565604	loss
nodBCC3	whole chr10			135534747	gain
nodBCC3	10	38990265	42680756	3690491	loss
nodBCC3	whole chr12			133851895	gain
nodBCC3	whole chrX			155270560	gain
nodBCC3	9	12091	68412833	68400742	gain
nodBCC3	9	68413199	141071710	72658511	loss
nodBCC4	14	64496749	107283160	42786411	CN_LOH
nodBCC4	whole chr15			102531392	loss
nodBCC5	10	42384457	42600232	215775	gain
nodBCC5	1	121484059	121485382	1323	gain
nodBCC5	16	46387396	46432728	45332	gain
nodBCC5	whole chrX			155270560	loss
nodBCC5	9	68410004	110093970	41683966	loss
nodBCC6	whole chr14			107349540	loss
nodBCC6	19	407900	24362490	23954590	loss

nodBCC6	3	239555	90306556	90067001	loss
nodBCC6	6	203433	58779318	58575885	gain
nodBCC6	8	182891	21890798	21707907	gain
nodBCC7	14	96761514	107283160	10521646	gain
nodBCC7	2	227729417	242842484	15113067	loss
nodBCC7	9	214706	69634684	69419978	gain
nodBCC7	9	71081186	141016262	69935076	loss
nodBCC8	whole chr2			243199373	gain
nodBCC8	7	193421	57659568	57466147	loss
nodBCC8	7	61968883	159025709	97056826	gain
nodBCC8	9	69002294	141016262	72013968	CN_LOH
nodBCC9	11	93212161	134241094	41028933	CN_LOH
nodBCC9	19	373897	9434930	9061033	loss
nodBCC9	19	12491498	24345092	11853594	loss
nodBCC9	2	204275861	242946576	38670715	gain
nodBCC9	3	386520	58641185	58254665	loss
nodBCC9	9	214779	21304803	21090024	gain
nodBCC9	9	21333254	38396065	17062811	loss
nodBCC9	9	71628207	141016262	69388055	loss
nodBCC10	14	45501562	107283178	61781616	gain
nodBCC10	9	16699302	141023998	124324696	loss
nodBCC10	22q			30000000	CN_LOH

copy neutral - LOH events across mBCC and nodBCC samples
