# Supplementary material for: GLI2 and FLNB Define Periocular Morphoeic Basal Cell Carcinoma
Source: Int J Mol Sci. 2025 Nov 25;26(23):11377. doi: 10.3390/ijms262311377 (PMC12692270; doi:10.3390/ijms262311377)
Supplement: Supplementary file 1 [file ijms-26-11377-s001.zip › Supplementary Table S6.pdf]

	WES			RNASeq			
**Pathway**	**Kegg ID**	**q-val**	**F**	**NES**	**GS**	**q-val**	**P-val**
Hedgehog_signaling_pathway	Hsa04340	7.23E-10	9	2.17	42	8.5E-04	<0.001
Basal_cell_carcinoma	Hsa05217	2.33E-08	9	2.30	46	<0.001	<0.001
Axon_guidance	Hsa04360	0.53	8	1.56	115	0.107	0.002
Natural_killer_cell_mediated_cytotoxicity	Hsa04650	0.042	8	-2.13	98	1.2E-04	<0.001
Fc_epsilon_ri_signaling_pathway	Hsa04664	0.07	6	-1.50	66	0.073	0.020

Supplementary Table S6. Correlation of mBCC WES and RNAseq pathway analysis. Intogen pathway prediction highlighting altered pathways using WES data from 10 mBCC tumours. MSigDB Pathway expression when comparing morphoeic tumour versus nodular tumour. WES; whole exome sequencing; Kegg ID, KEGG pathway identification number; q-val, q-value; F, frequency of mutations within tumour sample; NES, normalised enrichment score; GS, geneset size; p-val, P-value
