# Supplementary material for: GLI2 and FLNB Define Periocular Morphoeic Basal Cell Carcinoma
Source: Int J Mol Sci. 2025 Nov 25;26(23):11377. doi: 10.3390/ijms262311377 (PMC12692270; doi:10.3390/ijms262311377)
Supplement: Supplementary file 1 [file ijms-26-11377-s001.zip › Supplementary Table S7.pdf]

	WES			RNASeq			
**Pathway**	**ID**	**Q-val**	**F**	**NES**	**GS size**	**q-val**	**P-val**
Mismatch_repair	Hsa03430		5	1.84	22	0.069	0.002
Basal_cell_carcinoma	Hsa05217	2.25E-09	9	1.82	46	0.041	<0.001
TGFB	Hsa04350	1.2E-0.4	9	1.03	75	0.846	0.379
P53	Hsa04115	1.2E-0.4	10	-1.09	65	0.448	0.310
PPAR	Hsa03320	0.005	9	-1.54	51	0.053	0.011
Oxidative phosphorylation	Hsa00190	0.04	9	-2.41	108	<0.001	<0.001

Supplementary Table S7. Correlation of nodBCC WES and RNAseq pathway analysis. Intogen pathway prediction highlighting altered pathways using WES data from 10 nodBCC tumours. MSigDB Pathway expression when comparing morphoeic tumour versus nodular tumour. MSigDB, molecular signature database; Kegg ID, KEGG pathway identification number; q-val, q-value; F, frequency of mutations within tumour sample; NES, normalised enrichment score; GS, geneset size; p-val, P-value
