# Supplementary material for: GLI2 and FLNB Define Periocular Morphoeic Basal Cell Carcinoma
Source: Int J Mol Sci. 2025 Nov 25;26(23):11377. doi: 10.3390/ijms262311377 (PMC12692270; doi:10.3390/ijms262311377)
Supplement: Supplementary file 1 [file ijms-26-11377-s001.zip › Supplementary Table S8.pdf]

Supplementary Table S8

37 differentially expressed genes between morpheic stroma and nodular stroma comparison, using raw p < -0.01 and log2FC >1 or <-1

Ensembl_ID	Name	AveExpr	log2FC (morpheic stroma vs nodular stroma)	t	P.Value
ENSG000000	ABHD5	4.3996962	1.529624693	3.18441211	0.00659226
ENSG000001	LPXN	4.07428719	2.064805748	3.15807118	0.00694737
ENSG000001	CCR2	2.09944434	3.49448839	3.30405419	0.00519371
ENSG000002	AC009237.8	2.03248388	4.613868461	4.0295027	0.00123209
ENSG000001	GSTM1	1.1551889	7.618132726	7.95122247	1.43E-06
ENSG000000	ZNF446	1.12330168	3.066256951	3.04060505	0.00877654
ENSG000001	RSPH4A	0.93563461	3.978090582	3.07752121	0.00815544
ENSG000001	SIM2	0.86913417	3.388206046	3.06604854	0.00834365
ENSG000001	CD1B	0.53461448	4.236316116	3.13373248	0.00729235
ENSG000001	ACVR1C	0.47129134	4.920764449	3.73659684	0.00219623
ENSG000001	ZNF696	0.43375964	3.906542662	3.13082511	0.00733468
ENSG000002	7SK	0.24240576	4.166701995	3.14476516	0.00713391
ENSG000000	ABCA7	0.23319155	3.491316049	3.17530726	0.00671291
ENSG000002	RP11-484D2.	0.19413022	4.256937234	3.09687723	0.00784738
ENSG000001	SERPINA11	0.17578172	4.415548703	3.20612693	0.00631313
ENSG000001	ARL14	0.15745328	4.031815145	3.15588135	0.00697773
ENSG000002	AC005537.2	0.1062631	4.235647328	3.01511973	0.00923239
ENSG000001	DACT2	-0.0187543	3.678722118	2.99872199	0.00953798
ENSG000002	RP11-381E24	-0.0658663	4.352641184	3.20361018	0.00634487
ENSG000001	SMPD3	-0.3372442	3.427558035	3.24106724	0.0058885
ENSG000002	FGF14-AS2	-0.3419378	3.770641346	3.14922754	0.0070708
ENSG000001	DMRTA1	-0.3996042	-5.168823581	-3.3367501	0.00486597
ENSG000002	RPTN	-0.6292282	4.102415939	3.58939935	0.00294185
ENSG000002	OR7E62P	-0.6333857	4.010790214	3.03247415	0.00891951
ENSG000001	LAIR2	-0.6997905	3.306397012	2.99631269	0.00958371
ENSG000001	ST6GALNAC5	-0.7144842	5.030891615	2.9946475	0.00961544
ENSG000001	TMEM155	-0.7520662	4.278798323	3.50887922	0.0034531
ENSG000001	TMEM156	-0.9275787	3.866759454	2.98185538	0.00986271
ENSG000002	SSR4P1	-1.0099088	4.031701091	3.47732961	0.00367702
ENSG000002	ASS1P1	-1.1485764	3.663311482	3.45461454	0.00384723
ENSG000002	RP11-57K17.	-1.3756256	4.670416523	3.50264056	0.00349626
ENSG000002	LINC00299	-1.4317205	4.086604413	3.85318786	0.00174367
ENSG000002	CTD-2267D1!	-1.5018218	-3.505607892	-3.1240819	0.0074338
ENSG000002	RP11-96K19.	-1.63591	3.937349739	3.06738514	0.00832151
ENSG000001	FAM154A	-1.6412287	4.164536493	3.51495346	0.00341159
ENSG000002	RP11-296O1	-1.6455228	3.647588315	2.99899161	0.00953287
ENSG000002	RP13-15M17	-1.8328894	-4.071801721	-3.0886971	0.00797613
