# Supplementary material for: GLI2 and FLNB Define Periocular Morphoeic Basal Cell Carcinoma
Source: Int J Mol Sci. 2025 Nov 25;26(23):11377. doi: 10.3390/ijms262311377 (PMC12692270; doi:10.3390/ijms262311377)
Supplement: Supplementary file 1 [file ijms-26-11377-s001.zip › supplementary Table S9.pdf]

Upregulated pathways in mBCC stroma compared to nodBCC stroma

NAME	SIZE	ES	NES	NOM p-val
KEGG_PRIMARY_IMMUNODEFICIE	28	0.6683767	1.9954795	0
KEGG_T_CELL_RECEPTOR_SIGNA	99	0.5054894	1.81361	0
KEGG_ASTHMA	21	0.64456505	1.8039082	0
KEGG_ABC_TRANSPORTERS	40	0.53698754	1.7187824	0
KEGG_OLFACTORY_TRANSDUCTI	26	0.58066887	1.6782471	0.00235018
KEGG_NON_SMALL_CELL_LUNG_	51	0.5092922	1.6719804	0.00213904
KEGG_ALLOGRAFT_REJECTION	29	0.55202585	1.662966	0.00695249
KEGG_AUTOIMMUNE_THYROID_D	31	0.5399459	1.6417713	0.00675676
KEGG_NATURAL_KILLER_CELL_M	98	0.44667652	1.5945878	0.00102354
KEGG_INTESTINAL_IMMUNE_NET	35	0.4951832	1.5605984	0.01116072

Downregulated pathways in mBCC stroma compared to nodBCC stroma

NAME	SIZE	ES	NES	NOM p-val
KEGG_OXIDATIVE_PHOSPHORYLA	108	-0.64829	-3.6567209	0
KEGG_PARKINSONS_DISEASE	105	-0.6351795	-3.5291183	0
KEGG_HUNTINGTONS_DISEASE	158	-0.4690195	-2.77836	0
KEGG_RIBOSOME	85	-0.5164093	-2.5715423	0
KEGG_ALZHEIMERS_DISEASE	147	-0.4612289	-2.523712	0
KEGG_CARDIAC_MUSCLE_CONTR	61	-0.5014294	-2.3381412	0
KEGG_PROTEASOME	42	-0.525969	-2.3322227	0
KEGG_PYRUVATE_METABOLISM	32	-0.4741647	-1.9885563	0
KEGG_GLYCOLYSIS_GLUCONEOG	48	-0.3980002	-1.8817164	0
KEGG_RNA_POLYMERASE	28	-0.4500155	-1.8321027	0
KEGG_CYSTEINE_AND_METHIONI	29	-0.4139196	-1.7248966	0.00719425
KEGG_PROTEIN_EXPORT	24	-0.3924291	-1.5676357	0.02189781
KEGG_PROPANOATE_METABOLIS	31	-0.3255171	-1.4334846	0.0483871

Supplementary Table S9. GSEA significant upregulated and downregulated pathways in mBC

FDR q-val
0
0.01397647
0.01247175
0.03224223
0.04658219
0.04129057
0.04050497
0.04580558
0.07514113
0.10266209

FDR q-val
0
0
0
0
0
0
0
0.0031803
0.00615395
0.00953496
0.01587329
0.04765429
0.103025

thways in mBCC stroma compared to nodBCC stroma
